# Canine adipose tissue-derived MSCs engineered with mRNA to overexpress TSG-6 and enhance the anti-inflammatory effects in canine macrophages

**DOI:** 10.3389/fvets.2023.1134185

**Published:** 2023-04-06

**Authors:** Ga-Hee Yun, Su-Min Park, Ga-Hyun Lim, Kyoung-Won Seo, Hwa-Young Youn

**Affiliations:** Laboratory of Veterinary Internal Medicine, Department of Clinical Veterinary Science, College of Veterinary Medicine, Seoul National University, Seoul, Republic of Korea

**Keywords:** canine adipose tissue, mesenchymal stem cell, immunomodulation, TSG-6, *in vitro* transcription, mRNA, genetic engineering

## Abstract

**Background:**

Mesenchymal stem cells (MSCs) are useful agents in the treatment of various inflammatory diseases. The immunomodulatory effects of MSCs are largely related to their secretory properties. mRNA engineering emerged as a safe alternative to enhance the secretory function of MSCs. Optimization of the untranslated region (UTR) sequence is important for enhancing the translational efficiency of exogenous mRNAs. However, research on the optimization of UTR in canine MSCs has not yet been conducted.

**Objectives:**

We aimed to identify the UTR sequence related to the expression efficiency of *in vitro* transcription (IVT) mRNA in canine MSCs and investigate whether mRNA-engineered MSCs that overexpress TSG-6 exhibit enhanced anti-inflammatory effects.

**Methods:**

Canine adipose tissue-derived (cAT)-MSCs were transfected with *green fluorescence protein (GFP)* mRNA with three different UTRs: *canine hemoglobin subunit alpha-like 1 (HBA1), HBA2*, and *hemoglobin subunit beta-like (HBB)*. The translation efficacy of each mRNA was evaluated using relative fluorescence. *TSG-6* mRNA was produced with the UTR optimized according to relative fluorescence results. cAT-MSCs were transfected with *TSG-6* mRNA (MSC^TSG-6^), and TSG-6 expression was analyzed using real-time quantitative PCR, ELISA, and western blotting. To evaluate the anti-inflammatory effects of MSCs^TSG-6^, DH82 cells were co-cultured with MSCs^TSG-6^ or treated with dexamethasone, and changes in the expression of inflammatory cytokines were analyzed using qPCR.

**Results:**

The highest fluorescence level was observed in the *HBA1* UTR at 24 h post-transfection. *TSG-6* mRNA transfection yielded high levels of TSG-6 in the cAT-MSCs. In DH82 cells co-cultured with MSCs^TSG-6^, the expression of inflammatory cytokines decreased compared to that in co-culturing with naïve MSCs and dexamethasone treatment.

**Conclusions:**

Optimization of the *HBA1* UTR improved the translation efficiency of IVT mRNA in canine MSCs. cAT-MSCs engineered with *TSG-6* mRNA effectively enhanced the anti-inflammatory effects of the MSCs when co-cultured with LPS-activated DH82 cells.

## 1. Introduction

mRNA is a promising nucleic acid-based therapeutic agent with a wide range of applications, including vaccination and protein replacement therapies (1). Unlike DNA therapeutics, mRNA does not require nuclear entry because translation occurs in the cytoplasm and presents no risk of insertional mutagenesis because it does not integrate into the genome (2). mRNA is degraded *via* normal physiological pathways, resulting in low toxicity. Moreover, the production of mRNA through *in vitro* transcription (IVT) is rapid and inexpensive; therefore, mRNA-based therapeutics are considered a promising new drug class (3).

Despite these advantages, the application of exogenous mRNA is limited by instability and a short half-life. Modification of the structural elements of mRNA could resolve these issues, one approach being the optimization of the mRNA untranslated region (UTR) sequence (4). There are several regions in mRNA that affect translation efficacy and degradation by nuclease, including the 5′ cap, 5′- and 3′-UTRs, open reading frame (ORF), and poly (A) tail. Among these, 5′- and 3′-UTRs promote mRNA stability and translation efficacy (5). Several studies have shown that mRNA stability can be improved by modifying the UTR sequence of proteins: modification with human α- and β-globin UTR sequences promoted the amount and duration of protein production (6–8). mRNA-based therapeutics have since gained widespread acceptance, from vaccine and protein replacement therapies to cancer immunotherapy and stem cell genome engineering (9).

Because of their ability to modulate inflammatory responses, mesenchymal stem cells (MSCs) are promising treatment agents in various inflammatory diseases, such as inflammatory bowel disease (10), pancreatitis (11), rheumatoid arthritis (12), and peritonitis (13). MSCs exert their anti-inflammatory effects by secreting various paracrine soluble factors, among which tumor necrosis factor (TNF)-α-induced gene/protein 6 (TSG-6) is a major factor regulating inflammation in pancreatitis (14), lung injury (15, 16), inflammatory bowel disease (17), myocardial infarction (18), corneal injury (19), and skin wounds (20). One of the mechanisms underlying the immunomodulation of TSG-6 is its effect on macrophage polarization. Macrophages in the inflammatory context can be classified into two subsets: M1 and M2. Activated M1 macrophages promote inflammation by secreting inflammatory cytokines, such as interleukin (IL)-1β and TNF-α, whereas M2 macrophages secrete cytokines, such as IL-10, inducing an anti-inflammatory response (21). Recent studies have shown that MSCs alleviate inflammation by switching macrophages from the M1 to M2 phenotype *via* TSG-6 secretion (22, 23).

The clinical application of MSC therapies is limited by their insufficient secretion capacity. One solution is to genetically modify MSCs to overexpress specific therapeutic factors. Although viral-based gene delivery is commonly used, its clinical application is limited by safety concerns, such as genome insertion; therefore, mRNA transfection systems have emerged as a safe and effective alternative (24). MSCs engineered with mRNA to overexpress therapeutic factors, such as insulin growth factor-1 (IGF-1) and IL-10, showed enhanced anti-inflammatory action in osteoarthritis, acute graft-vs.-host disease, and traumatic brain injury models (25–27). Yang et al. (28) also improved the therapeutic effect of MSCs in a rat periodontitis model by overexpressing TSG-6 through viral-based engineering. However, application of an mRNA transfection system to increase TSG-6 expression in MSCs has not yet been reported for any animal model. Moreover, given the lack of research on UTR sequences for efficient mRNA expression in animal cell lines, including dogs, studies on mRNA-based therapeutics in veterinary medicine are urgently needed.

In this study, we aimed to (1) identify the UTR sequence regulating the expression efficiency of IVT mRNA in canine cell lines and (2) investigate whether mRNA-engineered MSCs that overexpress TSG-6 exhibit enhanced anti-inflammatory effects.

## 2. Materials and methods

### 2.1. DNA template design

The template of IVT mRNA consisted of the T7 promoter (5′-TAATACGACTCACTATAGGG-3′), 5′-UTR, ORF, and 3′-UTR. Three canine globin 5′- and 3′-UTRs were used to identify the UTR sequence that most effectively stabilized mRNA: canine *hemoglobin subunit alpha-like 1 (HBA1), HBA2*, and *hemoglobin subunit beta-like (HBB)*. The ORF was designed to encode green fluorescence protein (GFP) to evaluate expression efficiency with relative fluorescence, and a stop codon was placed downstream of the GFP-encoding sequence.

To produce *TSG-6* mRNA, the 5′- and 3′-UTRs of *HBA1* were used based on the relative fluorescence results. The designed DNA templates were synthesized using the Gene in Fragment form synthesis service provided by BIONICS (Korea). All the UTRs and ORF sequences were obtained from the National Center for Biotechnology Information (NCBI, USA).

### 2.2. *In vitro* transcription

Fragment DNA (0.2 μg) was used as a template for IVT. mRNA was produced using an mMESSAGE mMACHINE T7 ULTRA Transcription Kit (Life Technologies, Carlsbad, CA, USA) according to the manufacturer's instructions. Briefly, linear template DNA was transcribed with T7 enzyme and 5′ capped with Anti-Reverse Cap Analog (ARCA) during transcription; DNase I treatment and poly (A) tailing were then performed. The synthesized mRNA was purified using the lithium chloride (LiCl) precipitation method and quantified using a Nanophotometer Pearl (Implen, München, Germany).

### 2.3. Cell culture

Canine adipose tissue-derived (cAT)-MSCs were cultured in Dulbecco's modified Eagle's medium (DMEM; Welgene, Gyeongsan-si, Korea), supplemented with 10% fetal bovine serum (FBS; Gibco, Billings, MT, USA) and 1% penicillin/streptomycin (PS; PAN-Biotech, Aidenbach, Germany). DH82, a canine macrophage cell line, was purchased from the American Type Culture Collection (ATCC number: CRL-10389; Manassas, VA, USA) and maintained in DMEM with 15% FBS and 1% PS. All cells were incubated at 37°C and 5% CO_2_. The medium was changed every 2–3 days, and the cells were subcultured when they reached 70–80% confluence. cAT-MSCs from the third passage were used for all subsequent experiments.

### 2.4. Isolation and characterization of cAT-MSCs

Canine adipose tissues were obtained from three healthy adult female dogs undergoing ovariohysterectomy at the Seoul National University Veterinary Medical Teaching Hospital (SNU VMTH) under a protocol approved by the Institutional Animal Care and Use Committee (IACUC) of SNU (protocol no. SNU-220818-1). MSC isolation and culture were performed as previously described (29). Adipose tissue samples were washed with Dulbecco's phosphate-buffered saline (DPBS; Welgene) supplemented with 1% PS, disintegrated using sterilized scissors, treated with collagenase type IA (0.1%; Gibco), and incubated at 37°C for 1 h. After incubation, tissue sample neutralization was performed using DMEM supplemented with 10% FBS. The neutralized samples were centrifuged at 1,200 × *g* for 5 min, and the pellet was passed through a 70-μm cell strainer (Thermo Fisher Scientific, Waltham, MA, USA) and again centrifuged at 1,200 × *g* for 10 min. To remove the remaining red blood cells, the pellet was resuspended in RBC lysis buffer (Sigma-Aldrich, St. Louis, MO, USA), incubated at room temperature for 5 min, washed with DPBS, and centrifuged at 1,200 × *g* for 5 min. After removing the supernatant, the pellets were resuspended in DMEM with 10% FBS and 1% PS and seeded at a density of 3,000 cells/cm^2^ in 100 mm cell culture dishes. Cells were maintained at 37°C in 5% CO_2_ and the medium was replaced every 2–3 days. Subculture was performed when cells exhibiting fibroblast-like morphology reached 70–80% confluence.

To assess their differentiation capacities, cAT-MSCs were cultured using three differentiation media (StemPro Adipogenesis Differentiation, Stem Pro Osteogenesis Differentiation, and StemPro Chondrogenesis Differentiation kits; Gibco). Differentiation into adipocytes, osteocytes, and chondrocytes was identified by staining the cells with Oil Red O, 1% alizarin red, and alcian blue (Sigma-Aldrich), respectively. Additionally, the expression of specific surface markers was evaluated with flow cytometry using FACS Aria II system (BD Biosciences). Cells were stained with monoclonal antibodies against CD34-PE conjugated, CD45-FITC conjugated, CD29-FITC conjugated, and CD90-PE conjugated (BD Biosciences, San Jose, CA, USA), and the resulting fluorescence was analyzed using Flowjo software (Tree Star, Woodburn, OR, USA).

### 2.5. mRNA transfection in cAT-MSCs and effective UTR selection

We compared the translation efficacy of the *GFP* mRNAs with the UTRs *HBA1, HBA2*, and *HBB* in transfected cAT-MSCs. Cells (1 × 10^5^) were seeded in confocal dishes and transfected with 0.5 μg of each mRNA for 24 h with Lipofectamine MessengerMAX Reagent (Thermo Fisher Scientific) and Opti-MEM I Reduced Serum Medium (Gibco) according to the manufacturers' instructions. The fluorescence levels of transfected cells were measured using a confocal laser scanning microscope (LSM710; Zeiss, Germany) at 6, 12, 24, 36, 48, 60, 72, 84, and 96 h post-transfection. At each time point, three images per confocal dish were randomly acquired. The relative fluorescence was calculated using ImageJ software (National Institutes of Health, Bethesda, MD, USA). Briefly, we evaluated the area and intensity of green fluorescence using the ImageJ as the cells were evenly seeded. The fluorescence area was relatively calculated by assuming the total area as 1.

### 2.6. RNA extraction, cDNA synthesis, and real-time quantitative PCR (qPCR)

Total RNA of *TSG-6* mRNA-transfected cAT-MSCs and co-cultured DH82 cells was extracted using an Easy-Blue total RNA extraction kit (iNtRON Biotechnology, Seongnam, Korea). The concentration and the purity of samples were measured using a Nanophotometer Pearl (Implen). CellScript All-in-One 5 × 1st cDNA Strand Synthesis Master Mix (CellSafe, Bucheon-si, Korea) was used for cDNA synthesis. qPCR was conducted in duplicate using AMPIGENE qPCR Green Mix Hi-Rox with SYBR green dye (Enzo Life Sciences, Farmingdale, NY, USA), 400 nM forward and reverse primers, and 1 μL of cDNA. Thermocycler settings were 95°C for 2 min, followed by 40 cycles at 95°C for 5 s and 60°C for 25 s. Expression levels were analyzed using the 2^−ΔΔ/*Cts*^ method and normalized to that of *Glyceraldehyde 3-phosphate dehydrogenase (GAPDH)*. Primer sequences used in this study are listed in Table 1.

**Table 1:** Primer sequences for canine genes assessed in this study.

**Target gene**	**Primer**	**Sequence**	**Reference**	**Gene accession number**
*GAPDH*	Forward	TTA ACT CTG GCA AAG TGG ATA TTG T	(30)	NM_001003142.2
	Reverse	GAA TCA TAC TGG AAC ATG TAC ACC A		
*IL-6*	Forward	ATG ATC CAC TTC AAA TAG TCT ACC	(30)	NM_001003301.1
	Reverse	AGA TGT AGG TTA TTT TCT GCC AGT G		
*IL-1β*	Forward	AGT TGC AAG TCT CCC ACC AG	(31)	NM_001037971.1
	Reverse	TAT CCG CAT CTG TTT TGC AG		
*TNF-α*	Forward	TCA TCT TCT CGA ACC CCA AG	(30)	NM_001003244.4
	Reverse	ACC CAT CTG ACG GCA CTA TC		
*TSG-6*	Forward	TCC GTC TTA ATA GGA GTG AAA GAT G	(30)	XM_038426285.1
	Reverse	AGA TTT AAA AAT TCG CTT TGG ATC T		

### 2.7. Protein extraction and western blot analysis

Total protein of *TSG-6* mRNA-transfected cAT-MSCs was extracted using Pro-Prep protein extraction solution (iNtRON Biotechnology). The concentration of extracted protein was quantified using a DC Protein Assay Kit (Bio-Rad Laboratories, Hercules, CA, USA). We separated 20 μg of protein by SDS-PAGE, which was then transferred to polyvinylidene difluoride membranes (EMD Millipore, Burlington, MA, USA). The membranes were blocked with 5% non-fat dry milk and exposed to primary antibodies against TSG-6 (1:500, Santa Cruz Biotechnology, Santa Cruz, CA, USA) and β-actin (1:1,000, Santa Cruz Biotechnology) at 4°C for 24 h. Membranes were washed and then incubated with secondary antibodies at room temperature for 1 h. We detected the immunoreactive bands of the proteins using a chemiluminescence kit (Advansta, San Jose, CA, USA); the protein expression levels were normalized to that of β-actin.

### 2.8. Enzyme-linked immunosorbent assay (ELISA)

We measured TSG-6 content in the cell culture supernatant of *TSG-6* mRNA-transfected cAT-MSCs using a commercial TSG-6 ELISA kit (MyBioSource, San Diego, CA, USA) according to the manufacturer's instructions.

### 2.9. Co-culture with DH82 and TSG-6 mRNA transfected cAT-MSCs (MSC^*TSG*−6^)

cAT-MSCs (4 × 10^5^) were seeded onto 0.4 μM pore-sized Transwell inserts (SPL Life Sciences, Pocheon-si, Korea) and transfected with 2.5 μg *TSG-6* mRNA per well for 24 h (or without, as a control), using Lipofectamine MessengerMAX Reagent and Opti-MEM I Reduced Serum Medium. After 24 h, the inserts were washed twice with DPBS and the medium was changed to DMEM supplemented with 10% FBS and 1% PS.

DH82 cells were seeded in 6-well plates at a density of 1 × 10^6^ cells/well and incubated with or without 200 ng/mL lipopolysaccharide (LPS; Sigma-Aldrich) for 24 h. After changing the medium to DMEM with 10% FBS and 1% PS, DH82 macrophages were co-cultured with *TSG-6* mRNA-transfected cAT-MSCs, naïve cAT-MSCs, or treated with 10 μM dexamethasone (a well-known anti-inflammatory drug) for 24 h. This concentration of dexamethasone (24-h treatment) was selected because it effectively reduced inflammatory cytokine (IL-1β and IL-6) expression in LPS-stimulated DH82 cells without affecting viability, based on a Cell Counting Kit-8 (CCK-8) assay (Donginbio, Seoul, Korea) and qPCR (Supplementary Figure 1).

### 2.10. Statistical analysis

Each experiment was conducted at least three times. Data are shown as the mean ± standard deviation. Mean values from different groups were compared using the Mann–Whitney *t*-test and one-way analysis of variance using GraphPad Prism v.7.01 software (GraphPad Software, La Jolla, CA, USA). Statistical significance was set at *P* < 0.05.

## 3. Results

### 3.1. Characterization of cAT-MSCs

**Figure 1:**
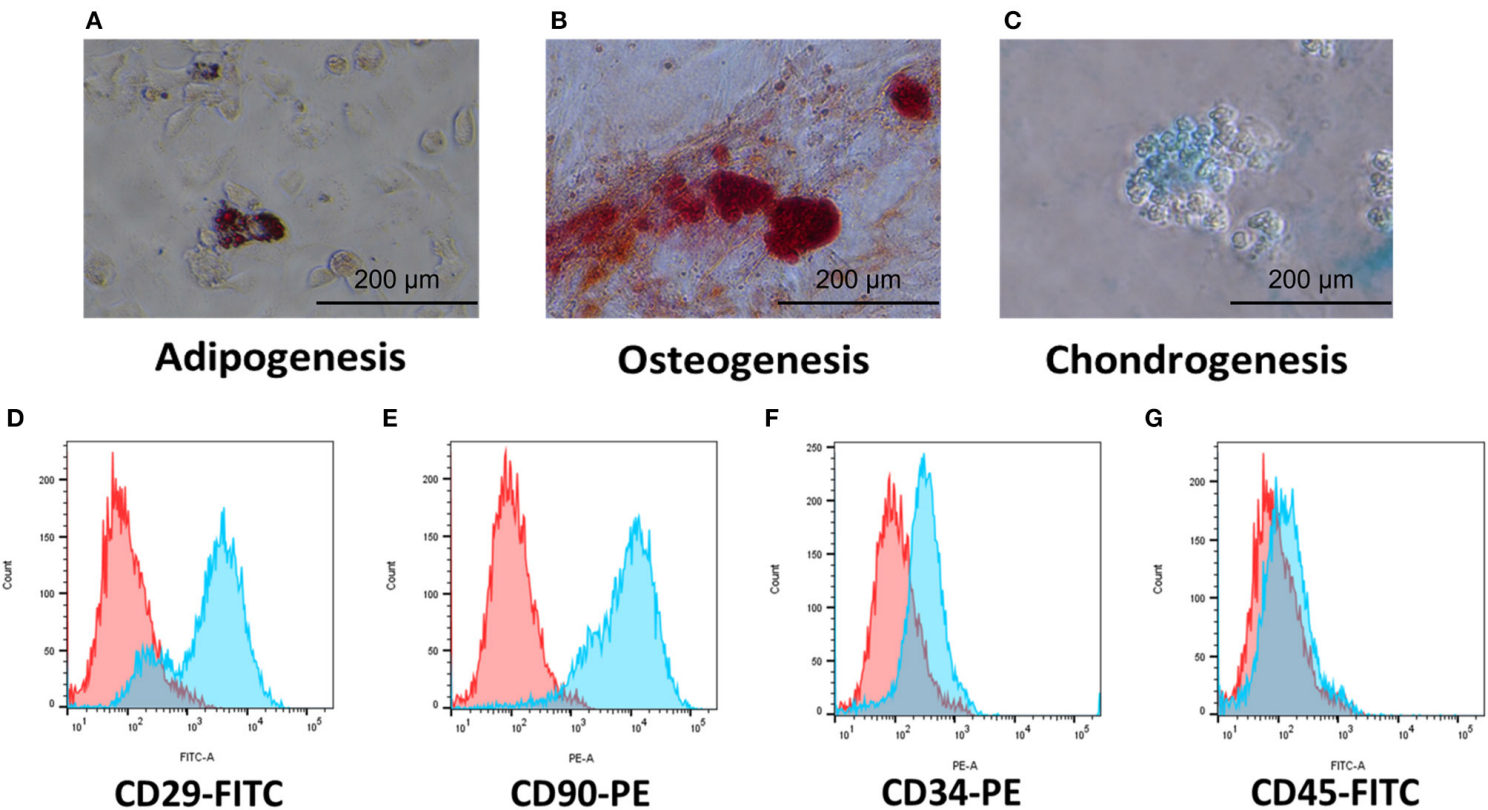
Characterization of cAT-MSCs isolated from canine adipose tissue. **(A–C)** Trilineage potential of cAT-MSCs. Differentiation into adipocyte, osteocyte, and chondrocyte was confirmed *via* Oil Red O, Alizarin Red, and Alcian Blue staining, respectively. **(D–G)** Flow cytometry analysis of cell surface markers. cAT-MSCs expressed CD29 and CD90 and lacked CD34 and CD45. (Blue: stained marker, red: unstained negative control).

We evaluated the stem cell identity of the cAT-MSCs using differentiation assays and flow cytometry. The cells were successfully differentiated into adipocytes, osteocytes, and chondrocytes (Figures 1A–C). Flow cytometry analysis showed high expression of the stem cell markers CD29 and CD90, while no expression of the hematopoietic markers CD34 and CD25 was detected (Figures 1D–G).

### 3.2. Efficient UTR selection in GFP mRNA-transfected cAT-MSCs

**Figure 2:**
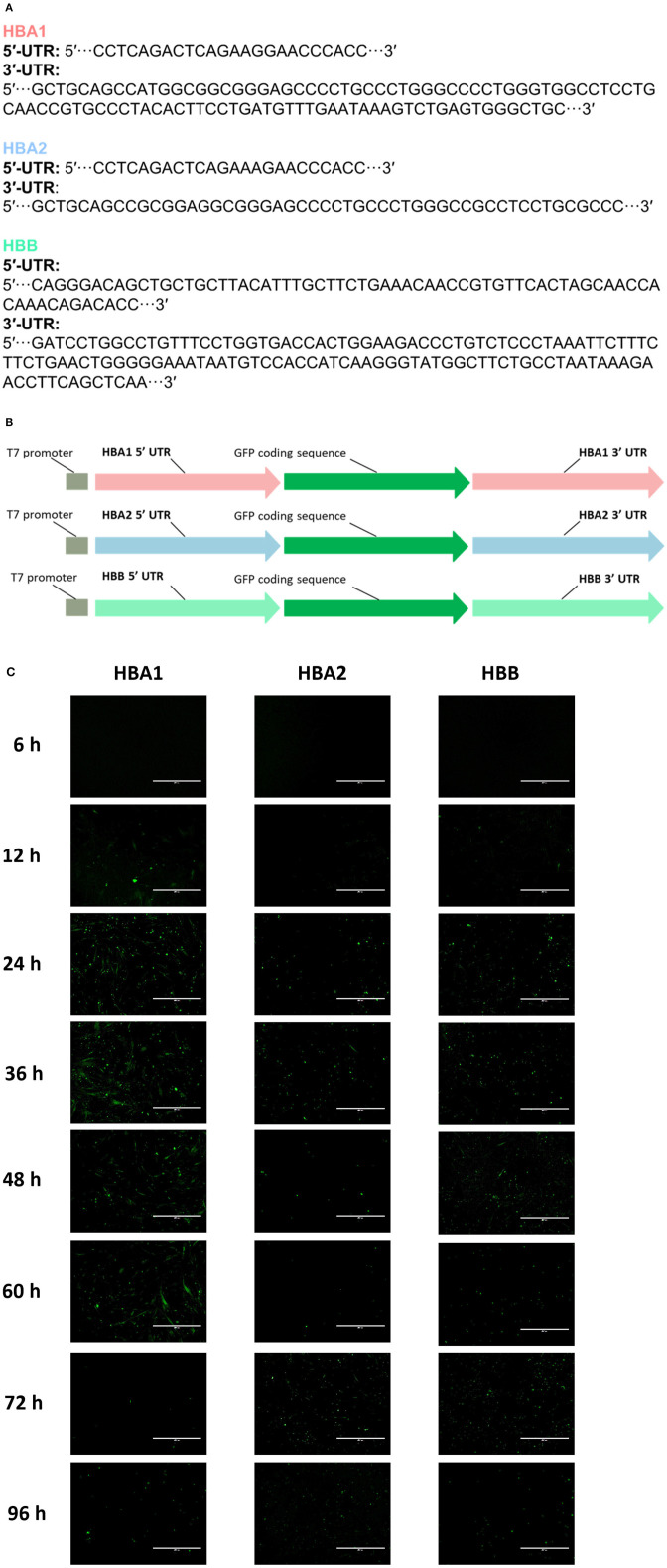
cAT-MSCs expressing GFP with three different UTRs. **(A)** Sequences of *HBA1, HBA2*, and *HBB* UTR regions. **(B)** Schematic structure of each template DNA, consisting of a T7 promoter, a 5′-UTR, a GFP coding region, and a 3′-UTR. **(C)** The highest fluorescence level was observed with *HBA1* 24 h post-transfection. *HBA2* and *HBB* reached peak fluorescence levels at 12 and 72 h post-transfection, both at lower levels than those of *HBA1*. Fluorescence was observed up to 96 h post-transfection. Scale bar, 400 μm (*HBA1*: *GFP* mRNA with UTR of canine hemoglobin subunit alpha-like 1; *HBA2*: *GFP* mRNA with UTR of canine hemoglobin subunit alpha-like 2; *HBB*: *GFP* mRNA with UTR of canine hemoglobin subunit beta-like).

**Figure 3:**
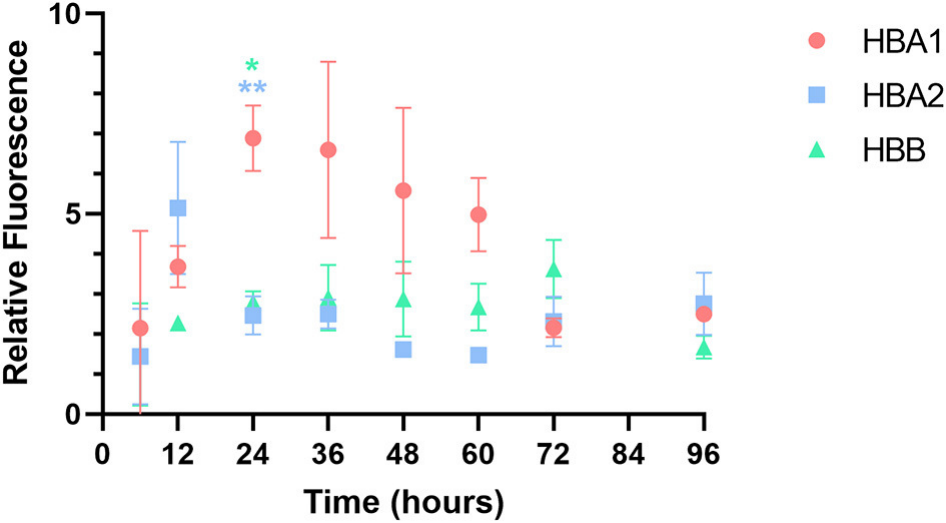
Relative fluorescence of each *GFP* mRNA over time. Among the three *GFP* mRNAs, *HBA1-GFP* mRNA showed the highest fluorescence level 24 h after transfection. Results are shown as the mean ± standard deviation. ^*^P < 0.05, ^**^P < 0.01 vs. *HBA2* and *HBB* at the same time point. (*HBA1*: *GFP* mRNA with UTR of canine hemoglobin subunit alpha-like 1; *HBA2*: *GFP* mRNA with UTR of canine hemoglobin subunit alpha-like 2; *HBB*: *GFP* mRNA with UTR of canine hemoglobin subunit beta-like).

cAT-MSCs were transfected with *GFP* mRNA containing three different UTRs (*HBA1, HBA2*, and *HBB*) and monitored for 96 h (Figures 2A, B). We observed significantly different fluorescence levels depending on the UTR, indicating variation in the translation efficacy of each mRNA. *HBA1*-, *HBA2*-, and *HBB*-*GFP* mRNA reached a peak fluorescence level at 24, 12, and 72 h, respectively, of which *HBA1* had the highest fluorescence level and *HBB* had the lowest (Figure 2C). At 24 h post-transfection, *HBA1* showed a significantly higher level of fluorescence than *HBA2* and *HBB* did. *HBA1* maintained a higher fluorescence level than the other two mRNAs for about 36 h. Fluorescence expression of *HBA1* was observed up to 96 h post-transfection (Figure 3). Overall, *HBA1* most effectively enhanced mRNA stability and translation efficacy.

### 3.3. Enhancement of TSG-6 expression in cAT-MSC^*TSG*−6^

**Figure 4:**
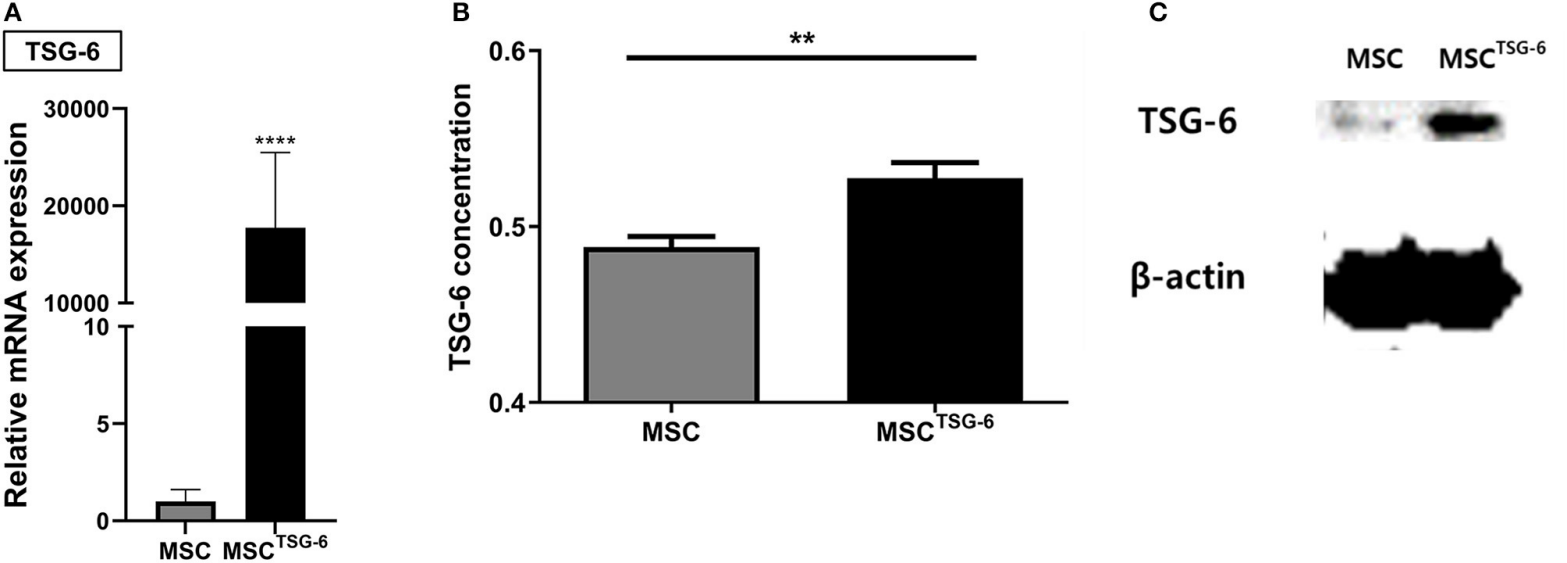
TSG-6 mRNA transfection increased expression of TSG-6 in cAT-MSCs. **(A)** TSG-6 mRNA level was increased in cAT-MSCs transfected with TSG-6 mRNA (MSC^TSG-6^). **(B)** TSG-6 protein level was increased in MSCs^TSG-6^. **(C)** Secretion of TSG-6 was also increased in MSCs^TSG-6^. Results are shown as the mean ± standard deviation. ^**^P < 0.01, ^****^P < 0.0001.

*TSG-6* mRNA was produced with the *HBA1* UTR and transfected into cAT-MSCs for 24 h. The coding sequence of *TSG-6* mRNA is described in Supplementary Data 1. The expression of *TSG-6* mRNA was more than 10,000-times higher in the MSC^TSG-6^ than in the MSC group, indicating the successful transfection of *TSG-6* mRNA (Figure 4A, Supplementary Tables 1, 2). Western blot confirmed that the expression of TSG-6 protein was significantly higher in the MSC^TSG-6^ group than that in the MSC group (Figure 4B). TSG-6 ELISA also confirmed that the secretion of TSG-6 was enhanced in the MSC^TSG-6^ group (Figure 4C). These results demonstrate that *TSG-6* mRNA transfection could produce high levels of TSG-6 in cAT-MSCs.

### 3.4. cAT-MSC^*TSG*−6^ co-culture decreases the macrophage-related expression of inflammatory cytokines

**Figure 5:**
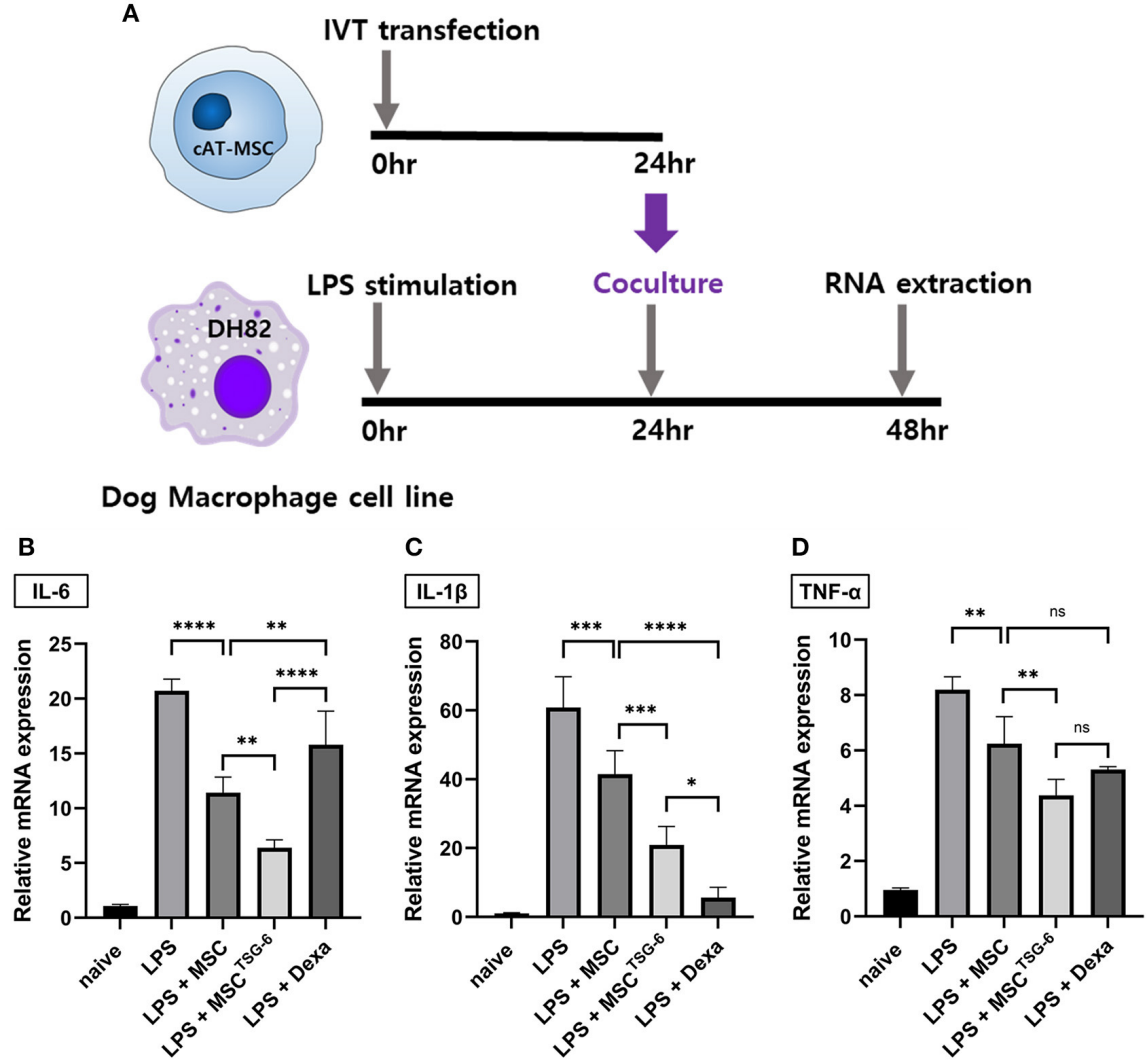
MSC^TSG-6^ co-culture altered the phenotype of DH82 macrophages. **(A)** Schematic diagram of co-culture with DH82 cells and MSCs^TSG-6^. **(B–D)** LPS treatment-induced M1 polarization of DH82 cells. IL-6, IL-1β, and TNF-α were used as M1 phase markers. The mRNA expression of these markers increased significantly in LPS-stimulated DH82 cells. Co-culture of these cells with naïve MSCs or MSCs^TSG-6^ reduced the mRNA level of M1 markers. MSCs^TSG-6^ more effectively reduced the expression of M1 markers than naïve MSCs did. **(B)** MSCs^TSG-6^ more effectively reduced IL-6 expression than dexamethasone did. **(C)** mRNA level of IL-1β showed a greater decrease with dexamethasone than MSCs^TSG-6^. **(D)** MSCs^TSG-6^ and dexamethasone reduced *TNF-*α mRNA expression to a comparable degree. Results are shown as the mean ± standard deviation obtained in three independent experiments. *P < 0.05, **P < 0.01, ***P < 0.001, ****P < 0.0001. ns, not significant.

We evaluated the immunomodulatory effect of MSC^TSG-6^ in the co-culture system with DH82 macrophages (Figure 5A). In LPS-stimulated DH82 cells, the mRNA expression of inflammatory cytokines demonstrating the M1 polarization significantly increased compared to the naïve group. In contrast, in DH82 cells co-cultured with MSCs or MSCs^TSG-6^, the expression of inflammatory cytokines was significantly decreased compared with that in the LPS-stimulated group. Compared to the MSC-co-cultured group, the mRNA expression levels of all inflammatory cytokines were significantly lower in the MSC^TSG-6^-co-cultured group (Figures 5B–D, Supplementary Tables 3–6).

The anti-inflammatory effect of MSC^TSG-6^ was compared with that of dexamethasone. We compared the expression levels of inflammatory cytokines between LPS-stimulated DH82 cells treated with 10 μM dexamethasone to those co-cultured with MSCs or MSCs^TSG-6^. The concentration of IL-6 was higher in the dexamethasone-treated group than in the MSC- and MSC^TSG-6^-co-cultured groups (Figure 5B). In contrast, the mRNA expression level of *IL-1*β was lower in the dexamethasone-treated group than in the MSC- and MSC^TSG-6^-co-cultured groups (Figure 5C). *TNF-*α mRNA expression levels did not vary between groups (Figure 5D).

## 4. Discussion

Through their secretion of immunomodulatory factors, such as TSG-6, MSCs show promising therapeutic effects against various inflammatory diseases (32). However, challenges related to the survival and secretion efficacy of MSCs have hindered their clinical application. Varied strategies, such as biochemical priming and genetic engineering, have been applied to enhance the secretory functions of MSCs (33). In this study, we investigated whether the immunomodulatory effects of canine MSCs can be enhanced by *TSG-6* mRNA transfection.

First, we generated three different GFP-encoding mRNAs using the UTRs of canine *HBA1, HBA2*, and *HBB*, and evaluated their expression efficacy to identify the UTR optimized for canine MSCs. The highest peak fluorescence intensity level was observed with *HBA1-GFP* mRNA, and its relative fluorescence intensity was higher than that for *HBA2-* and *HBB-GFP* for ~36 h. Fluorescence expression of *HBA1-GFP* mRNA was observed up to 96 h post-transfection. Given that the half-life of wild-type GFP is 26 h (34), our findings indicated that UTR optimization increased mRNA stability.

This is the first study to attempt UTR optimization to improve IVT mRNA translation efficiency in canine MSCs. Holtkamp et al. (35) demonstrated that the effect of the UTR sequence on mRNA translation efficiency depends on the cell type. This indicates that UTR optimization should be designed according to the target cells. In human cells, the UTR of globin, with a half-life of 16–48 h, is commonly used for UTR optimization to increase mRNA stability (36). The mechanism of mRNA stabilization is related to the low G-C content (~44%) of the 5′-UTR of human β-globin, which reduces the likelihood that a secondary structure will be formed and inhibit protein translation (37). In addition, the pyrimidine-rich element (PRE) in the 3′-UTR of human β-globin is a recognition site for RNA-binding proteins that regulate mRNA stability (38). In the present study, the canine *HBA1* UTR improved the stability of mRNA and expression of TSG-6. Although further studies are needed to determine whether canine globin UTR enhances mRNA stability by the same mechanism as human globin, our findings can inform the design of suitable templates to increase the translation efficiency of IVT mRNA in canine MSCs.

Pro-inflammatory cytokine treatment and viral engineering have also been used to modify the secretion of anti-inflammatory factors by MSCs. Several studies have demonstrated that priming MSCs with pro-inflammatory cytokines, such as TNF-α and IFN-γ, effectively increases their immunosuppressive properties (39). However, preconditioning with pro-inflammatory cytokines carries a risk of toxicity, even at low concentrations (40). In addition, priming MSCs with pro-inflammatory cytokines induces non-specific increase in the secretion of overall factors. Depending on the disease context, some factors secreted may be helpful, but some factors may also be detrimental (39). Viral engineering of MSCs can induce expression more accurately and stably than other preconditioning methods but harbors the risk of genetic mutation and tumorigenicity (41). In contrast, engineering MSCs with mRNA only induces overexpression of specific therapeutic factor and does not have any risk of genetic mutation. Therefore, our study presents a reduced-risk genetic engineering method to effectively modulate TSG-6 secretion and anti-inflammatory effects.

We produced TSG-6-overexpressing canine MSCs by transfecting cAT-MSCs with *TSG-6* mRNA and found that, compared to naïve MSCs, the MSCs^TSG-6^ caused a greater reduction in inflammatory cytokine expression in LPS-stimulated DH82 cells. We also compared the anti-inflammatory effects of MSC^TSG-6^ with those of dexamethasone. Although 10 μM dexamethasone was a higher concentration than that reported to effectively lower the expression of inflammatory cytokines in macrophage cells (42, 43), the degree of decrease in the expression of IL-6 and TNF-α in the MSC^TSG-6^ co-culture group was higher or similar to that with 10 μM dexamethasone. These results indicate that TSG-6, induced by MSC mRNA engineering, has an anti-inflammatory effect comparable to that of dexamethasone, which is consistent with the findings of previous studies of topically administered TSG-6 vs. systemic dexamethasone (44, 45). Dexamethasone is a potent anti-inflammatory and immunosuppressive drug that is widely used in the treatment of various inflammatory and autoimmune diseases, but its long-term use is limited due to its systemic side effects on endocrine and metabolic functions (46). However, MSC engineering through mRNA transfection induces relatively transient target protein overexpression, so the risk of long-term side effects is low (26). In this respect, our results that MSC^TSG-6^ showed a similar anti-inflammatory effect to dexamethasone could be noteworthy. For future clinical application, evaluating the long-term therapeutic efficacy and side effects of dexamethasone and MSC^TSG-6^ in various inflammatory disease models is necessary.

We acknowledge the limitations of our study. First, UTR optimization was performed using the ORF of *GFP*. It is not clear whether this optimized UTR can be applied to other ORFs, as different ORFs can affect the secondary structure and the resulting translation efficiency of mRNA (4). In addition, an *in vivo* study to evaluate the therapeutic efficacy of *TSG-6* mRNA-engineered MSC in inflammatory disorders is needed. Nevertheless, our study is the first to successfully optimize the UTR of canine MSCs and genetically engineer canine MSCs *via* mRNA transfection. Our study provides an empirical basis for mRNA-based therapeutics, including MSC engineering, in veterinary medicine, and our framework could be applied to designing IVT mRNA-encoding therapeutic factors other than TSG-6.

## Data availability statement

The original contributions presented in the study are included in the article/Supplementary material, further inquiries can be directed to the corresponding author.

## Ethics statement

The animal study was reviewed and approved by Institutional Animal Care and Use Committee (IACUC) of Seoul National University (SNU). Written informed consent was obtained from the owners for the participation of their animals in this study.

## Author contributions

G-HY and S-MP contributed to conception and design of the study, organization of data, statistical analysis, and manuscript writing. G-HL performed the organization of data and statistical analysis. K-WS supervised and reviewed the manuscript. H-YY designed and supervised the study. All authors contributed to manuscript revision, read, and approved the submitted version.
