# Supplementary material for: Canine adipose tissue-derived MSCs engineered with mRNA to overexpress TSG-6 and enhance the anti-inflammatory effects in canine macrophages
Source: Front Vet Sci. 2023 Apr 6;10:1134185. doi: 10.3389/fvets.2023.1134185 (PMC10118014; doi:10.3389/fvets.2023.1134185)
Supplement: Supplementary file 1 [file Data_Sheet_1.docx]

Supplementary Material

Canine Adipose Tissue-Derived MSCs Engineered with mRNA to Overexpress TSG-6 Enhance the Anti-Inflammatory Effect in Canine Macrophages

Hwa-Young Youn*, Ga-Hee Yun, Su-Min Park, Ga-Hyun Lim, Kyoung-Won Seo

*** Correspondence:** Hwa-Young Youn: hyyoun@snu.ac.kr

# Supplementary Figures

**
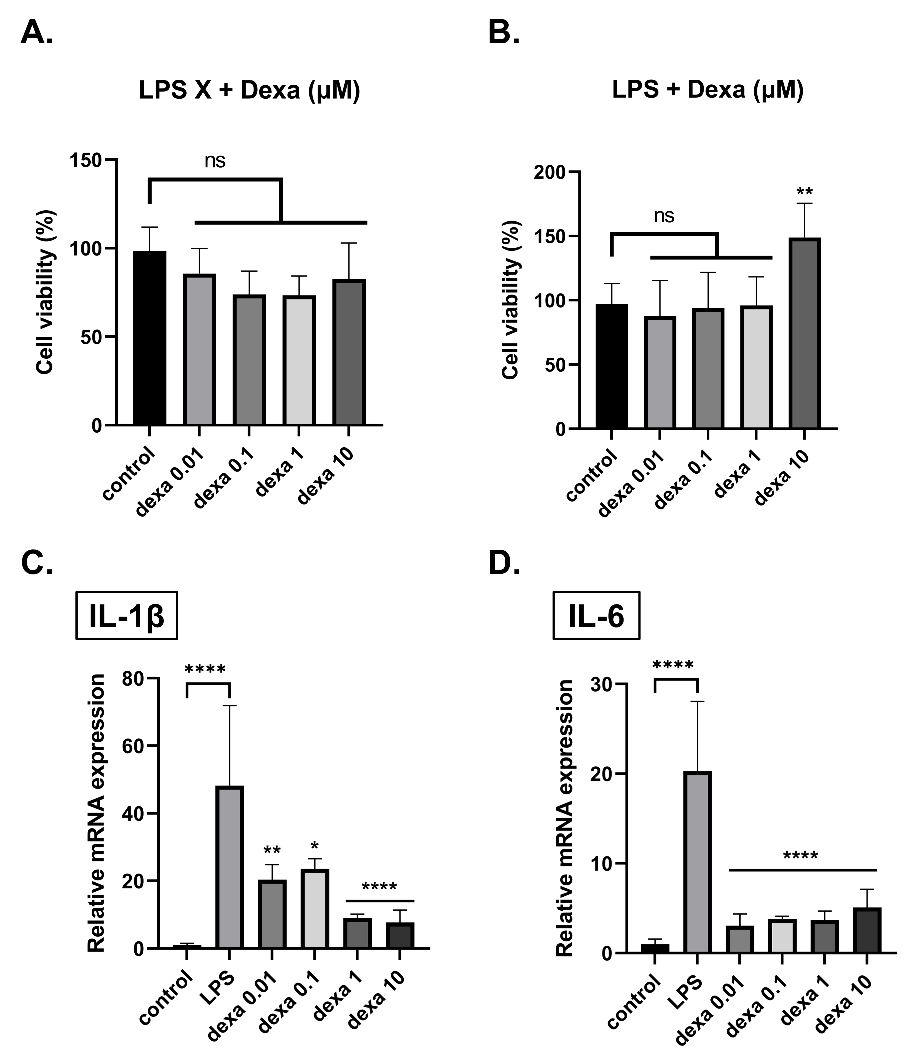
**

**Supplementary Figure 1.** **Viability and inflammatory cytokine expression of DH82 macrophages treated with dexamethasone.**

**(A, B)** Treatment of naïve or LPS-stimulated DH82 macrophage cells with dexamethasone. **(C, D)** IL-1β and IL-6 expression of LPS-stimulated DH82 macrophage cells treated with dexamethasone. Treatment with 10 μM dexamethasone reduced the expression of inflammatory cytokines significantly without cytotoxicity. Results are shown as the mean ± standard deviation. *P < 0.05, **P < 0.01 versus control (b) or LPS (c), ****P < 0.0001. ns, not significant.

# Supplementary Data

**Coding sequence of *TSG-6* mRNA**

The coding sequence (CDS) of *TSG-6* mRNA was obtained from NCBI (XM_038426285.1;

ATGATCATCTTTATTTCCTTATTTGTCTTGCTGTGGGAAGAGGCGCACGGATGGGGATTCAACAATGGGATTTTTCATAACTCTATATGGCTTGAACAGGCAGCTGGCGTGTACCACCGGGAAGCTCGCTTGGGCCGCTACAGGCTCACGTACGCCGAGGCCAAGGCCGTGTGCGAGTTCGAGGGCGGCCGGCTCGCCACCTACAAGCAGCTGGAGGCGGCCCGCAAAATCGGATTTCACGTCTGCGCTGCTGGGTGGATGGCCAAGGGCAGAGTTGGATACCCCATTGTGAAACCGGGGCCCAACTGTGGATTTGGAAAAACTGGTATTATTGATTATGGAGTCCGTCTTAATAGGAGTGAAAGATGGGATGCCTATTGCTACAACCCACATGCAAAGGAGTGTGGTGGAGTCTTTACAGATCCAAAGCGAATTTTTAAATCTCCAGGCTTCCCAAATGAGTATGATGATAACCAGATCTGCTACTGGCACATTAGAGTCAAGTATGGTCAGCGTGTTCACCTGAGCTTTCTGGACTTTGACCTTGAGGATGACCCAGCCTGCTTGGCTGACTATGTTGAAATATATGACAGTTACGATGACGTTCATGGTTTTGTGGGAAGGTACTGTGGAGATGAGCTTCCAGAAGACATCATTAGTACAGGAAATGTCATGACCTTGAAGTTTCTAAGTGACGCTTCAGTGACAGCGGGAGGTTTCCAAATCAAGTACATTGCAGTGGATCCTCTATCCAAATCCAGTCAAGGAAAAAACACAAGCACTACTTCTACTGGAAATAAGAACTTTTTAGCTGGAAGATTTAG).

# Supplementary Tables

**Supplementary Table 1.** **C_T_ values for GAPDH and TSG-6 assessed by qPCR in MSCs**

| Sample | Sample number | C_T_ values of GAPDH | C_T_ values of TSG-6 | C_T_ of TSG-6 - C_T_ values of GAPDH | | |
| --- | --- | --- | --- | --- | --- | --- |
|  |  |  |  | **Values** | **Average** | **Standard deviation** |
| MSC | 1 | 19.868 | 27.488 | 7.6209526062012 | 7.221 | 0.766998 |
|  | 1 | 19.741 | 27.183 | 7.4413356781006 |  |  |
|  | 1 | 21.322 | 27.848 | 6.5258693695068 |  |  |
|  | 2 | 19.431 | 27.475 | 8.0448627471924 |  |  |
|  | 2 | 20.616 | 27.774 | 7.1577720642090 |  |  |
|  | 2 | 21.217 | 27.002 | 5.7852230072021 |  |  |
|  | 3 | 19.385 | 27.124 | 7.7386379241943 |  |  |
|  | 3 | 19.426 | 27.461 | 8.0347137451172 |  |  |
|  | 3 | 20.261 | 26.899 | 6.6376609802246 |  |  |
| MSC^TSG-6^ | 1 | 20.409 | 13.536 | -6.8732786178589 | -6.988 | 0.561225 |
|  | 1 | 20.344 | 13.962 | -6.3820610046387 |  |  |
|  | 1 | 21.391 | 13.304 | -8.0874385833740 |  |  |
|  | 2 | 21.345 | 15.089 | -6.2553939819336 |  |  |
|  | 2 | 21.153 | 14.357 | -6.7963848114014 |  |  |
|  | 2 | 21.328 | 13.950 | -7.3784990310669 |  |  |
|  | 3 | 20.516 | 13.386 | -7.1301927566528 |  |  |
|  | 3 | 20.393 | 13.701 | -6.6915884017944 |  |  |
|  | 3 | 20.559 | 13.260 | -7.2989797592163 |  |  |

**Supplementary Table 2. Melt curve result for GAPDH and TSG-6 in MSCs**

| Target name | Sample | Sample number | Tm | Melt Peak Height |
| --- | --- | --- | --- | --- |
|  |  |  |  |  |
| GAPDH | MSC | 1 | 83.651 | 47,754.395 |
|  |  | 1 | 83.344 | 44,608.063 |
|  |  | 1 | 84.265 | 25,466.195 |
|  |  | 2 | 83.037 | 44,671.043 |
|  |  | 2 | 84.419 | 40,028.977 |
|  |  | 2 | 84.572 | 44,112.609 |
|  |  | 3 | 83.190 | 43,166.719 |
|  |  | 3 | 83.190 | 42,543.633 |
|  |  | 3 | 84.112 | 42,319.410 |
|  | MSC^TSG-6^ | 1 | 20.409 | 46,555.770 |
|  |  | 1 | 20.344 | 43,575.246 |
|  |  | 1 | 21.391 | 44,368.805 |
|  |  | 2 | 21.345 | 46,631.992 |
|  |  | 2 | 21.153 | 45,764.523 |
|  |  | 2 | 21.328 | 43,883.391 |
|  |  | 3 | 20.516 | 43,957.008 |
|  |  | 3 | 20.393 | 42,322.516 |
|  |  | 3 | 20.559 | 36,609.031 |
| TSG-6 | MSC | 1 | 81.654 | 32,316.158 |
|  |  | 1 | 81.808 | 30,113.352 |
|  |  | 1 | 82.883 | 33,042.789 |
|  |  | 2 | 82.269 | 33,551.117 |
|  |  | 2 | 82.422 | 37,923.223 |
|  |  | 2 | 81.808 | 31,869.426 |
|  |  | 3 | 81.501 | 35,361.609 |
|  |  | 3 | 81.962 | 35,387.656 |
|  |  | 3 | 81.501 | 34,099.324 |
|  | MSC^TSG-6^ | 1 | 82.115 | 38,364.660 |
|  |  | 1 | 82.115 | 36,053.898 |
|  |  | 1 | 81.501 | 30,120.807 |
|  |  | 2 | 82.417 | 38,325.340 |
|  |  | 2 | 81.803 | 38,815.176 |
|  |  | 2 | 81.343 | 33,416.344 |
|  |  | 3 | 81.957 | 36,552.297 |
|  |  | 3 | 82.110 | 37,154.949 |
|  |  | 3 | 81.496 | 34,789.250 |

**Supplementary Table 3.** **C_T_ values for GAPDH and IL-6 assessed by qPCR in DH82 cells**

| Sample | Sample number | C_T_ values of GAPDH | C_T_ values of IL-6 | C_T_ of IL-6 - C_T_ values of GAPDH | | |
| --- | --- | --- | --- | --- | --- | --- |
|  |  |  |  | **Values** | **Average** | **Standard deviation** |
| naive | 1 | 19.204 | 33.783 | 14.5783882141113 | 14.046274 | 0.316955 |
|  | 1 | 19.116 | 33.163 | 14.0471267700195 |  |  |
|  | 2 | 20.297 | 34.168 | 13.8713188171386 |  |  |
|  | 2 | 20.396 | 34.589 | 14.1927413940429 |  |  |
|  | 3 | 19.910 | 33.563 | 13.6528835296630 |  |  |
|  | 3 | 20.172 | 34.107 | 13.9351882934570 |  |  |
| LPS | 1 | 19.571 | 29.329 | 9.7582473754883 | 9.7491525 | 0.177136 |
|  | 1 | 19.700 | 29.316 | 9.6158828735352 |  |  |
|  | 2 | 19.711 | 29.299 | 9.5887813568115 |  |  |
|  | 2 | 19.688 | 29.727 | 10.0394039154052 |  |  |
|  | 3 | 20.363 | 30.230 | 9.8668460845947 |  |  |
|  | 3 | 20.220 | 29.846 | 9.6257534027100 |  |  |
| LPS  +  MSC | 1 | 19.205 | 30.553 | 11.3475246429443 | 10.48258 | 0.599335 |
|  | 1 | 20.291 | 29.795 | 9.5043506622314 |  |  |
|  | 2 | 19.762 | 30.302 | 10.5397491455078 |  |  |
|  | 2 | 19.732 | 30.405 | 10.6733455657958 |  |  |
|  | 3 | 18.969 | 29.530 | 10.5605964660644 |  |  |
|  | 3 | 19.416 | 29.686 | 10.2699623107910 |  |  |
| LPS  +  MSC^TSG-6^ | 1 | 19.995 | 31.246 | 11.2510738372802 | 11.261542 | 0.208607 |
|  | 1 | 19.827 | 31.113 | 11.2863903045654 |  |  |
|  | 2 | 19.843 | 31.044 | 11.2011108398437 |  |  |
|  | 2 | 19.717 | 31.330 | 11.6129112243652 |  |  |
|  | 3 | 19.211 | 30.467 | 11.2557487487792 |  |  |
|  | 3 | 19.266 | 30.228 | 10.9620227813720 |  |  |
| LPS  +  Dexa | 1 | 19.561 | 29.394 | 9.8326778411865 | 9.6166807 | 1.26003 |
|  | 1 | 19.364 | 28.404 | 9.0406398773193 |  |  |
|  | 2 | 19.239 | 33.783 | 8.1114292144775 |  |  |
|  | 2 | 18.839 | 33.163 | 8.7294826507568 |  |  |
|  | 3 | 19.612 | 34.168 | 11.5925083160400 |  |  |
|  | 3 | 20.158 | 34.589 | 10.3933467864990 |  |  |

**Supplementary Table 4. C_T_ values for GAPDH and IL-1β assessed by qPCR in DH82 cells**

| Sample | Sample number | C_T_ values of GAPDH | C_T_ values of IL-1β | C_T_ of IL-1β - C_T_ values of GAPDH | | |
| --- | --- | --- | --- | --- | --- | --- |
|  |  |  |  | **Values** | **Average** | **Standard deviation** |
| naive | 1 | 19.204 | 29.307 | 10.1027355194091 | 9.826966 | 0.32513 |
|  | 1 | 19.116 | 29.258 | 10.1426277160644 |  |  |
|  | 2 | 20.297 | 30.383 | 10.0855674743652 |  |  |
|  | 2 | 20.396 | 30.102 | 9.7059593200684 |  |  |
|  | 3 | 19.910 | 29.425 | 9.5153827667236 |  |  |
|  | 3 | 20.172 | 29.581 | 9.4095268249512 |  |  |
| LPS | 1 | 19.571 | 23.418 | 3.8474521636963 | 3.882181 | 0.21932 |
|  | 1 | 19.700 | 23.452 | 3.7522907257080 |  |  |
|  | 2 | 19.711 | 23.618 | 3.9076805114746 |  |  |
|  | 2 | 19.688 | 23.288 | 3.6000175476074 |  |  |
|  | 3 | 20.363 | 24.619 | 4.2562599182129 |  |  |
|  | 3 | 20.220 | 24.149 | 3.9293880462646 |  |  |
| LPS  +  MSC | 1 | 19.205 | 23.323 | 4.1176490783691 | 4.136603 | 0.55216 |
|  | 1 | 20.291 | 23.444 | 3.1532840728760 |  |  |
|  | 2 | 19.762 | 24.361 | 4.5990219116211 |  |  |
|  | 2 | 19.732 | 24.330 | 4.5983791351318 |  |  |
|  | 3 | 18.969 | 23.397 | 4.4276618957520 |  |  |
|  | 3 | 19.416 | 23.340 | 3.9236259460449 |  |  |
| LPS  +  MSC^TSG-6^ | 1 | 19.995 | 24.988 | 4.9931163787842 | 5.139703 | 0.55254 |
|  | 1 | 19.827 | 25.146 | 5.3193111419678 |  |  |
|  | 2 | 19.843 | 25.619 | 5.7762184143066 |  |  |
|  | 2 | 19.717 | 25.415 | 5.6980991363525 |  |  |
|  | 3 | 19.211 | 23.706 | 4.4946708679199 |  |  |
|  | 3 | 19.266 | 23.823 | 4.5568027496338 |  |  |
| LPS  +  Dexa | 1 | 19.561 | 26.146 | 6.5855617523193 | 6.296807 | 1.9804 |
|  | 1 | 19.364 | 26.387 | 7.0229740142822 |  |  |
|  | 2 | 19.239 | 22.214 | 2.9755535125732 |  |  |
|  | 2 | 18.839 | 24.073 | 5.2339630126953 |  |  |
|  | 3 | 19.612 | 28.023 | 8.4109477996826 |  |  |
|  | 3 | 20.158 | 27.998 | 7.8406009674072 |  |  |

**Supplementary Table 5. C_T_ values for GAPDH and TNF-α assessed by qPCR in DH82 cells**

| Sample | Sample number | C_T_ values of GAPDH | C_T_ values of TNF-α | C_T_ of TNF-α - C_T_ values of GAPDH | | |
| --- | --- | --- | --- | --- | --- | --- |
|  |  |  |  | **Values** | **Average** | **Standard deviation** |
| naive | 1 | 19.204 | 28.233 | 9.02917098999023 | 8.904245 | 0.18893 |
|  | 1 | 19.116 | 28.168 | 9.05241394042968 |  |  |
|  | 2 | 20.297 | 29.323 | 9.02629661560058 |  |  |
|  | 2 | 20.396 | 29.356 | 8.95938110351562 |  |  |
|  | 3 | 19.910 | 28.693 | 8.78302955627441 |  |  |
|  | 3 | 20.172 | 28.747 | 8.57517814636230 |  |  |
| LPS | 1 | 19.571 | 25.493 | 5.92217445373535 | 6.03807 | 0.282301 |
|  | 1 | 19.700 | 25.451 | 5.75132179260253 |  |  |
|  | 2 | 19.711 | 25.556 | 5.84527778625488 |  |  |
|  | 2 | 19.688 | 25.612 | 5.92449188232421 |  |  |
|  | 3 | 20.363 | 26.729 | 6.36617469787597 |  |  |
|  | 3 | 20.220 | 26.639 | 6.41897964477539 |  |  |
| LPS  +  MSC | 1 | 19.205 | 25.808 | 6.60260009765625 | 6.190166 | 0.51062 |
|  | 1 | 20.291 | 25.525 | 5.23442268371582 |  |  |
|  | 2 | 19.762 | 26.226 | 6.46383476257324 |  |  |
|  | 2 | 19.732 | 26.033 | 6.30129432678222 |  |  |
|  | 3 | 18.969 | 25.484 | 6.51470375061035 |  |  |
|  | 3 | 19.416 | 25.440 | 6.02414321899414 |  |  |
| LPS  +  MSC^TSG-6^ | 1 | 19.995 | 26.369 | 6.37465858459472 | 6.3528006 | 0.39724 |
|  | 1 | 19.827 | 26.158 | 6.33102416992187 |  |  |
|  | 2 | 19.843 | 26.748 | 6.90583801269531 |  |  |
|  | 2 | 19.717 | 26.352 | 6.63484191894531 |  |  |
|  | 3 | 19.211 | 25.310 | 6.09942817687988 |  |  |
|  | 3 | 19.266 | 25.037 | 5.77101325988769 |  |  |
| LPS  +  Dexa | 1 | 19.561 | 26.032 | 6.47079086303710 | 6.594903 | 0.17519 |
|  | 1 | 19.364 | 25.878 | 6.51421356201171 |  |  |
|  | 2 | 19.239 | 25.691 | 6.45261955261230 |  |  |
|  | 2 | 18.839 | 25.600 | 6.76144218444824 |  |  |
|  | 3 | 19.612 | 26.481 | 6.86858367919921 |  |  |
|  | 3 | 20.158 | 26.659 | 6.50177383422851 |  |  |

**Supplementary Table 6. Melt curve result for GAPDH, IL-6, IL-1β, and TNF-α in DH82 cells**

| Target name | Sample | Sample number | Tm | Melt Peak Height |
| --- | --- | --- | --- | --- |
|  |  |  |  |  |
| GAPDH | Naïve | 1 | 83.012 | 49,784.320 |
|  |  | 1 | 83.317 | 41,093.852 |
|  |  | 2 | 83.164 | 40,423.711 |
|  |  | 2 | 83.012 | 44,620.742 |
|  |  | 3 | 82.866 | 42,721.828 |
|  |  | 3 | 82.713 | 42,684.137 |
|  | LPS | 1 | 83.018 | 39,733.387 |
|  |  | 1 | 83.018 | 37,541.898 |
|  |  | 2 | 83.164 | 38,768.258 |
|  |  | 2 | 82.554 | 44,797.703 |
|  |  | 3 | 83.621 | 50,857.641 |
|  |  | 3 | 83.316 | 51,948.453 |
|  | LPS + MSC | 1 | 83.469 | 45,926.547 |
|  |  | 1 | 84.231 | 41,718.391 |
|  |  | 2 | 83.622 | 39,271.527 |
|  |  | 2 | 82.860 | 40,696.859 |
|  |  | 3 | 82.866 | 46,389.234 |
|  |  | 3 | 83.933 | 41,305.902 |
|  | LPS + MSC^TSG-6^ | 1 | 82.713 | 39,992.164 |
|  |  | 1 | 82.713 | 36,252.664 |
|  |  | 2 | 82.859 | 40,693.309 |
|  |  | 2 | 83.316 | 41,851.598 |
|  |  | 3 | 83.164 | 44,379.766 |
|  |  | 3 | 82.859 | 49,988.902 |
|  | LPS + Dexa | 1 | 83.164 | 39,370.531 |
|  |  | 1 | 82.860 | 37,359.223 |
|  |  | 2 | 83.469 | 33,179.164 |
|  |  | 2 | 83.469 | 33,416.473 |
|  |  | 3 | 82.713 | 45,914.711 |
|  |  | 3 | 83.628 | 35,682.125 |
| IL-6 | Naïve | 1 | 84.384 | 28,934.686 |
|  |  | 1 | 83.622 | 36,738.707 |
|  |  | 2 | 83.774 | 35,579.477 |
|  |  | 2 | 84.079 | 47,153.918 |
|  |  | 3 | 83.476 | 37,214.359 |
|  |  | 3 | 83.933 | 37,067.816 |
|  | LPS | 1 | 84.238 | 35,841.316 |
|  |  | 1 | 83.476 | 33,254.789 |
|  |  | 2 | 83.773 | 48,821.961 |
|  |  | 2 | 84.383 | 40,613.734 |
|  |  | 3 | 84.535 | 35,567.703 |
|  |  | 3 | 83.926 | 40,716.492 |
|  | LPS + MSC | 1 | 84.841 | 29,005.180 |
|  |  | 1 | 83.926 | 40,052.844 |
|  |  | 2 | 83.926 | 34,360.770 |
|  |  | 2 | 84.231 | 31,012.117 |
|  |  | 3 | 83.476 | 37,332.648 |
|  |  | 3 | 83.933 | 34,786.781 |
|  | LPS + MSC^TSG-6^ | 1 | 84.085 | 33,023.824 |
|  |  | 1 | 84.238 | 32,324.463 |
|  |  | 2 | 83.468 | 38,355.426 |
|  |  | 2 | 83.773 | 39,810.508 |
|  |  | 3 | 84.535 | 32,903.930 |
|  |  | 3 | 83.316 | 35,619.273 |
|  | LPS + Dexa | 1 | 83.622 | 32,492.588 |
|  |  | 1 | 83.926 | 33,153.117 |
|  |  | 2 | 84.079 | 37,803.762 |
|  |  | 2 | 84.231 | 31,952.531 |
|  |  | 3 | 84.238 | 35,372.355 |
|  |  | 3 | 83.628 | 29,648.516 |
| IL-1β | Naïve | 1 | 87.432 | 29,950.828 |
|  |  | 1 | 87.127 | 37,741.293 |
|  |  | 2 | 86.974 | 36,786.125 |
|  |  | 2 | 86.517 | 31,156.174 |
|  |  | 3 | 86.524 | 34,270.758 |
|  |  | 3 | 87.592 | 31,090.420 |
|  | LPS | 1 | 85.000 | 41,647.203 |
|  |  | 1 | 86.220 | 38,053.313 |
|  |  | 2 | 85.450 | 42,100.570 |
|  |  | 2 | 86.059 | 40,477.012 |
|  |  | 3 | 86.212 | 40,877.090 |
|  |  | 3 | 84.840 | 48,817.852 |
|  | LPS + MSC | 1 | 86.212 | 37,090.449 |
|  |  | 1 | 85.146 | 43,250.688 |
|  |  | 2 | 85.146 | 44,546.188 |
|  |  | 2 | 84.993 | 52,157.164 |
|  |  | 3 | 85.000 | 44,243.242 |
|  |  | 3 | 85.000 | 32,172.418 |
|  | LPS + MSC^TSG-6^ | 1 | 85.000 | 43,918.270 |
|  |  | 1 | 85.305 | 43,546.027 |
|  |  | 2 | 86.059 | 38,292.695 |
|  |  | 2 | 85.602 | 52,157.004 |
|  |  | 3 | 85.755 | 45,856.277 |
|  |  | 3 | 86.059 | 58,571.801 |
|  | LPS + Dexa | 1 | 85.762 | 37,371.477 |
|  |  | 1 | 87.134 | 40,173.906 |
|  |  | 2 | 85.145 | 38,022.535 |
|  |  | 2 | 86.364 | 44,749.656 |
|  |  | 3 | 87.583 | 41,305.590 |
|  |  | 3 | 85.907 | 37,469.906 |
| TNF-α | Naïve | 1 | 83.475 | 58,066.695 |
|  |  | 1 | 83.475 | 51,367.949 |
|  |  | 2 | 83.475 | 50,728.387 |
|  |  | 2 | 83.475 | 46,807.805 |
|  |  | 3 | 83.175 | 60,572.695 |
|  |  | 3 | 83.175 | 59,446.176 |
|  | LPS | 1 | 83.328 | 60,939.320 |
|  |  | 1 | 83.023 | 45,713.145 |
|  |  | 2 | 83.469 | 60,328.266 |
|  |  | 2 | 83.622 | 57,651.664 |
|  |  | 3 | 84.536 | 63,161.223 |
|  |  | 3 | 84.536 | 61,681.184 |
|  | LPS + MSC | 1 | 84.847 | 48,708.660 |
|  |  | 1 | 83.627 | 31,784.988 |
|  |  | 2 | 83.475 | 50,373.023 |
|  |  | 2 | 83.322 | 47,219.195 |
|  |  | 3 | 83.328 | 63,824.402 |
|  |  | 3 | 83.633 | 54,184.617 |
|  | LPS + MSC^TSG-6^ | 1 | 83.785 | 51,196.516 |
|  |  | 1 | 83.785 | 57,116.023 |
|  |  | 2 | 83.469 | 61,228.750 |
|  |  | 2 | 83.927 | 51,159.145 |
|  |  | 3 | 84.536 | 57,080.695 |
|  |  | 3 | 83.317 | 67,698.438 |
|  | LPS + Dexa | 1 | 83.475 | 51,531.566 |
|  |  | 1 | 84.085 | 49,423.504 |
|  |  | 2 | 84.237 | 52,363.617 |
|  |  | 2 | 83.475 | 27,200.617 |
|  |  | 3 | 83.785 | 57,924.328 |
|  |  | 3 | 83.785 | 42,628.715 |
